# NLRP3 Inflammasome Activation Is Involved in Geniposide-Induced Hepatotoxicity

**DOI:** 10.1155/mi/4112856

**Published:** 2025-02-03

**Authors:** Yixuan Liu, Baoyue Liu, Mingzhu Shi, Tianxiang Ye, Huifang Li

**Affiliations:** School of Chinese Medicine and Food Engineering, Shanxi University of Traditional Chinese Medicine, Jinzhong 030619, China

**Keywords:** *Gardenia jasminoides* J. Ellis, geniposide, hepatotoxicity, NLRP3

## Abstract

**Background:** Geniposide, a prominent iridoid glycoside derived from *Gardenia jasminoides J*. Ellis, has garnered attention due to its association with hepatotoxicity despite its well-documented pharmacological efficacy in preclinical and clinical contexts. The NOD-like receptor protein 3 (NLRP3) inflammasome is implicated in numerous pathological conditions, including drug-induced liver injury. This study aims to explore the involvement of the NLRP3 inflammasome in geniposide-induced liver toxicity.

**Methods:** Rats were administered geniposide for 5 days, concurrently treated with or without glibenclamide (GLY), an in vivo inhibitor of NLRP3. In vitro, HL-7702 cells were exposed to genipin (a metabolite of geniposide via hepatointestinal circulation), with or without GLY supplement. Liver tissue was examined through pathological sections. Aspartate aminotransferase (AST), alanine aminotransferase (ALT), alkaline phosphatase (ALP), γ-glutamyl transpeptidase (γ-GT), and total bilirubin (T-Bil) levels were determined using the enzyme plate method. IL-1*β* and IL-18 levels in the supernatant and serum were quantified through ELISA. Apoptosis-associated speck-like protein (ASC), NLRP3, caspase-1, pro-IL-1*β*, and pro-IL-18 mRNA levels in cells or the liver were assessed by RT-PCR. Protein levels of ASC, NLRP3, caspase-1, pro-IL-1*β*, and pro-IL-18 in cells or the liver were analyzed by Western blot.

**Results:** Rats treated with geniposide displayed notable liver damage characterized by inflammatory infiltration, elevated serum transaminases, and heightened levels of inflammatory factors IL-1*β* and IL-18. This liver damage was concomitant with NLRP3 inflammasome activation within the liver. Furthermore, genipin induction led to reduced cell viability, increased transaminases in the cell supernatant, and an upsurge in inflammatory factors, resulting in heightened NLRP3 inflammasome expression within the cells. However, GLY effectively curtailed excessive NLRP3 activation, dampened the production of inflammatory factors IL-1*β* and IL-18, and ameliorated liver damage caused by geniposide.

**Conclusions:** Our findings collectively elucidate that geniposide induces hepatotoxicity by triggering NLRP3 inflammasome signaling. Inhibition of the inflammasome presents a promising novel therapeutic target for mitigating geniposide-induced hepatotoxicity.

## 1. Introduction

In recent years, there has been a significant increase in the usage of traditional Chinese medicine intreatment of diseases and dietary supplements globally. A recent survey indicates that a majority of individuals consider traditional Chinese medicine to be harmless [1]. However, an alarming event occurred in the 1990s when kidney failure was linked to the use of Aristolochia, a traditional Chinese medicine. Subsequent research has unveiled the potential for traditional Chinese medicine to inflict damage on various organs, especially the liver [2]. As a result, the issue of hepatotoxicity related to traditional Chinese medicine is attracting considerable attention.

Fructus Gardeniae (FG), the dried ripe fruit of *Gardenia jasminoides J*. Ellis (Rubiaceae), is a classic Chinese herb used both for medicinal and culinary purposes. In the realm of traditional Chinese medicine, FG is believed to influence the heart, lung, and triple burner meridians, with the ability to quell excessive internal heat, alleviate restlessness, and cool the blood [3]. Historically, it has been employed to treat conditions such as febrile diseases accompanied by restlessness, damp-heat jaundice, and mycotic stomatitis [4], and it is documented in the Chinese pharmacopeia for its capacity to dispel internal heat, ease mental distress, reduce fever, induce diuresis, and cool the blood to alleviate pathogenic heat [5]. Additionally, in China, FG is used in food recipes and dietary supplements, often mixed with tea or included in health foods.

Geniposide, the primary bioactive compound found in FG, is renowned for its array of pharmacological functions, including neuroprotection, antidiabetic properties, hepatoprotection, anti-inflammatory action, analgesic effects, cardioprotection, and various other beneficial attributes. It is widely utilized in the treatment of diverse conditions such as jaundice, headaches, and diarrhea, owing to its anti-inflammatory and hepatoprotective capabilities [6, 7]. Recent animal studies have unveiled FG extracts induced hepatotoxicity in rats [8, 9],and histopathological examination of rat liver tissue has corroborated these toxic effects, demonstrating a correlation between increasing geniposide dosageand degree of hepatotoxicity [10]. As a consequence, a growing body of researchers posits that geniposide is the fundamental cause of FG's toxicity. Conversely, some scholars contend that genipin, formed from geniposide through hydrolysis by *β*-glucosidase in the intestine, possesses high biocompatibility and is the principal hepatotoxic component of FG [9, 11].Given its significant role as a bioactive component for treating hepatitis, it is imperative to investigate the mechanisms underlying its hepatotoxicity [12].

The NOD-like receptor protein 3 (NLRP3) inflammasome is an intracellular multiprotein complex characterized by a C-terminal leucine-rich domain, an N-terminal pyrin domain (PYD), and a nucleotide-binding domain [13]. When activated, the NLRP3 inflammasome recruits pro-caspase-1 and apoptotic speck-like protein via the PYD to form a complex which leads to the cleavage of caspase-1 and the maturation of interleukin-18 (IL-18) and interleukin-1*β* (IL-1*β*). These cytokines play a pivotal role in promoting liver inflammation [14, 15]. Aberrant NLRP3 activity is a contributing factor to the progression of inflammatory diseases, encompassing diabetes, Alzheimer's disease, and atherosclerosis [16, 17]. To date, several drugs have proved to induce NLRP3 inflammasome activation, leading to inflammatory injury and hepatotoxicity, including atorvastatin, lipopolysaccharide, and alcohol [12, 18]. Nevertheless, the involvement of NLRP3 inflammasome activation in geniposide toxicity and the precise mechanisms remain poorly understood.

Therefore, the primary aim of this study is to delineate the involvement of the NLRP3 inflammasome in geniposide-induced hepatotoxicity and elucidate the underlying mechanisms of their interaction. It is anticipated that the findings of this investigation will furnish a scientific foundation for the medical advancement of *G. jasminoides* Ellis.

## 2. Materials and Methods

### 2.1. Chemical Agents

Geniposide (HPLC purity > 99%, catalog no. 170312) was purchased from Shanghai Winherb Medical Science Co. Ltd. Genipin (HPLC purity > 99%, catalog no. 117C022) was procured from Beijing Solarbio Science & Technology Co., Ltd. Glibenclamide (GLY, HPLC purity > 98%, catalog no. 10438568) was obtained from Shanghai Macklin Biochemical Co. Ltd.

### 2.2. Animals and Grouping

Sprague Dawley rats, consisting of an equal number of males and females and weighing between 220 and 250 g, were obtained from Vital River Co. Ltd. (License No. SCXK (Jing) 2021–0011; Beijing, China). These rats were accommodated in a laboratory animal facility with controlled climate conditions, featuring a 12/12-h light/dark cycle for a duration of 7 days. The ambient temperature was regulated within the range of 22°C–26°C, and the relative humidity was adjusted to 60 ± 10%. Prior to the experiment, all rats were subjected to an overnight fast with unrestricted access to water, followed by a 12-h fast preceding euthanasia and necropsy. All procedures were conducted in accordance with the Regulations of Experimental Animal Administration, as published in 2019 by the State Committee of Science and Technology of the People's Republic of China. Every effort was exerted to reduce the number of animals used and mitigate their distress.

After an 1-week acclimatization period, 48 rats were randomly assigned into four groups, each containing 12 rats, as follows: Group C (control group), Group GE (model group, 450 mg/kg geniposide), Group GE + GLY (450 mg/kg geniposide and 40 mg/kg GLY), and Group GLY (40 mg/kg GLY). Each morning at 8:00, geniposide (450 mg/kg) was administered via gavage to Groups GE and GE + GLY, while the other groups received an equivalent volume of normal saline. After a 4-h interval, GLY (40 mg/kg) was administered via gavage to Groups GE + GLY and GLY, with the remaining groups receiving an equivalent volume of normal saline.

After 5 days of oral administration of the drugs, the rats were deprived of food for 24 h prior to the final treatment. On the day of killing, the rats were anesthetized using an intraperitoneal injection of 3% pentobarbital sodium. Blood was then collected from the abdominal aorta, anticoagulated with heparin sodium, and the livers were excised, weighed, and subsequently fixed in 10% neutral-buffered formalin. The livers were prepared for histopathological analysis by embedding in paraffin and staining sections with hematoxylin and eosin.

### 2.3. Cells and Grouping

Human normal liver cells (HL-7702) were cultured in RPMI-1640 medium containing 10% fetal bovine serum (FBS), 100 mM HEPES, 100 U/mL penicillin, and 100 μg/mL streptomycin. These cells were maintained at 37°C in a humidified 5% CO_2_ cell culture incubator.

HL-7702 cells were seeded in 96-well culture plates at a density of 1 × 10^6^ cells/mL and were divided into four groups as follows: Group C (control group), Group GN (model group, 200 μg/mL genipin), Group GN + GLY (200 μg/mL genipin and 100 μM GLY), and Group GLY (100 μM GLY). Following a 24-h drug treatment regimen, the supernatants were harvested and preserved at −80°C for subsequent analysis using enzyme-linked immunosorbent assay (ELISA), as well as for the determination of alanine aminotransferase (ALT), aspartate aminotransferase (AST), alkaline phosphatase (ALP), and *γ*-glutamyl transpeptidase (*γ*-GT) levels. The cell extracts were also stored at −80°C for Western blot and quantitative real-time PCR analyses.

### 2.4. Cytotoxicity Assay

Cell viability was assessed using CCK-8. Cells were plated in 96-well plates at a density of 5000 cells per well, following the cell grouping described above. CCK-8 reagent was added to each well and incubated for 1 h. Absorbance (OD) was measured at 450 nm using an automated enzyme labeler. Cell viability (%) was calculated using the formula: (OD of compound-treated cells −OD of CCK-8/OD of control-treated cells − OD of CCK-8) × 100%.

### 2.5. Histopathological Evaluation of the Liver

To evaluate liver inflammation and hepatotoxicity, hematoxylin and eosin staining were employed. Liver tissue samples were fixed by immersing the slides in 4% neutral formalin buffer for 24 h, followed by dehydration in graded ethanol. The tissue samples were then embedded in paraffin and sliced. Subsequently, the sections were stained with hematoxylin and eosin (HE), and the histopathological changes in liver tissue were examined under a microscope.

### 2.6. Biochemical Indicators Assay

Serum collected by centrifugation was utilized to measure ALT, AST, ALP,*γ*-GT, and total bilirubin (T-Bil) using commercial kits (Nanjing Jiancheng Biological Engineering Research Institute, Nanjing, China) and a Clinical Biochemistry Analyzer Chemray800 (RLAS Ltd., Shenzhen, China). Supernatant from cell cultures, obtained by centrifugation, were also used for measurement of ALT, AST, ALP, and *γ*-GT. The samples were excluded if hemolysis was observed in the blood specimen.

### 2.7. Enzyme-Linked Immunosorbent Assay

Supernatant from cell culture medium and rat sera was collected by centrifugation and used for the measurement of interleukin-1*β* (IL-1*β*; Raybiotech Life, Inc., GA, USA) and interleukin-18 (IL-18; R&D Systems, Inc., MND, USA) using ELISA kits in accordance with the manufacturer's protocols.

### 2.8. Quantitative Real-Time PCR

Quantitative real-time PCR was conducted following the manufacturer's instructions. Total RNA was extracted from the liver samples and cell extracts using a total RNA extraction kit (Tiangen biotech CO., Ltd., Beijing, China). cDNA was synthesized from the total RNA using FastKing-RT Enzyme (Tiangen biotech CO., Ltd., Beijing, China) as per the manufacturer's instructions. Real-time PCR was carried out with each primer using the FastFire qPCR PreMix (SYBR Green) (Tiangen biotech CO., Ltd., Beijing, China). The primer sequences for animals are listed in Table 1, while the primer sequences for cells are presented in Table 2.The PCR setting comprised an initial denaturation at 95°C for 1 min, followed by 40 cycles of denaturation at 95°C for 5 s, and annealing at 60°C for 15 s. The results were normalized to GAPDH mRNA expression level, and the fold change (2−ΔΔ*C*_t_) was compared with that of the control group.

### 2.9. Western Blotting

Total protein was extracted using RIPA lysis buffer (Solarbio, Beijing, China), supplemented with 1% protease inhibitor (Solarbio, Beijing, China). A total of 20 μg of protein was loaded onto a 10% SDS polyacrylamide gel and subjected to electrophoresis. Subsequently, the protein sample was transferred to polyvinylidene difluoride membranes (PVDFs; Millipore, Billerica, MA, USA). Blocking was performed with 5% skim milk for 1 h at room temperature to prevent nonspecific binding. This was followed by primary antibody incubation at 4°C for 12 h. The primary antibodies used in this study included anti-*β*-actin (1:1000 dilution; cat no. GB15003, Servicebio Biotechnology Co., Ltd. Wuhan, China), anti-NLRP3 (1:1000 dilution; cat no. bos8040bp4594, Boster Biological Technology. Co., Ltd. Wuhan, China), anti-apoptosis-associated speck-like protein (ASC) (1:2000 dilution; cat no. GB115270, Servicebio Biotechnology Co., Ltd. Wuhan, China), anti-pro-IL-1*β* (1:1000 dilution; cat no. AB9722, Abcam, Waltham, MA, USA), and anti-pro-IL−18 (1:1000 dilution; cat no. #10663-1-ap, Proteintech Group Co., Ltd. Wuhan, China). Subsequently, the membranes were probed with horseradish peroxidase-conjugated secondary antibodies (1:5000 dilution; cat no. 2205049, Servicebio Biotechnology Co., Ltd. Wuhan, China) at room temperature for 2 h. Membranes were incubated with ECL reagent (Servicebios), and GeneGnome XRQ (Gene Co., Ltd. Shanghai, China) was employed to detect protein signals. Image J software was utilized for protein quantification.

### 2.10. Statistical Analysis

Statistical analysis was conducted using GraphPad Prism software, SPSS (2007, IBM Inc., CA, USA). Differences among groups were assessed using one-way analysis of variance (ANOVA), followed by the post hoc “LSD Test” for comparisons involving more than two groups. Results are expressed as the mean ± standard deviation (SD). Statistical significance was established when the *p*-value < 0.05.

The study was conducted in accordance with the Basic and Clinical Pharmacology and Toxicology policy for experimental and clinical studies

## 3. Results

### 3.1. Cellular Vitality

**Figure 1:**
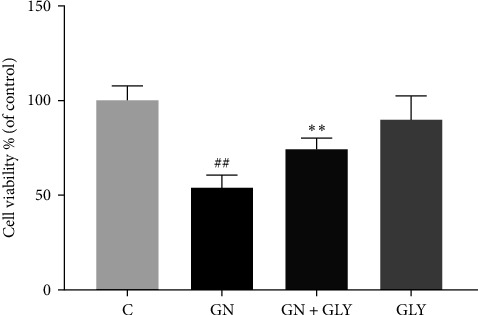
The effect of genipin on cell viability, *n* = 6(^#^*p* < 0.05, ^##^*p* < 0.01, vs., control group; *⁣*^*∗*^*p* < 0.05, *⁣*^*∗∗*^*p* < 0.01, vs., geniposide or genipin group). GN, genipin; GLY, glibenclamide.

The outcomes of the CCK8 assay indicate that genipin at a concentration of 200 µg/mL suppresses cellular proliferation. However, cotreatment with genipin and GLY enhances cellular viability, suggesting that GLY may mitigate the toxicity of genipin (Figure 1).

### 3.2. Biochemical Indicators

**Figure 2:**
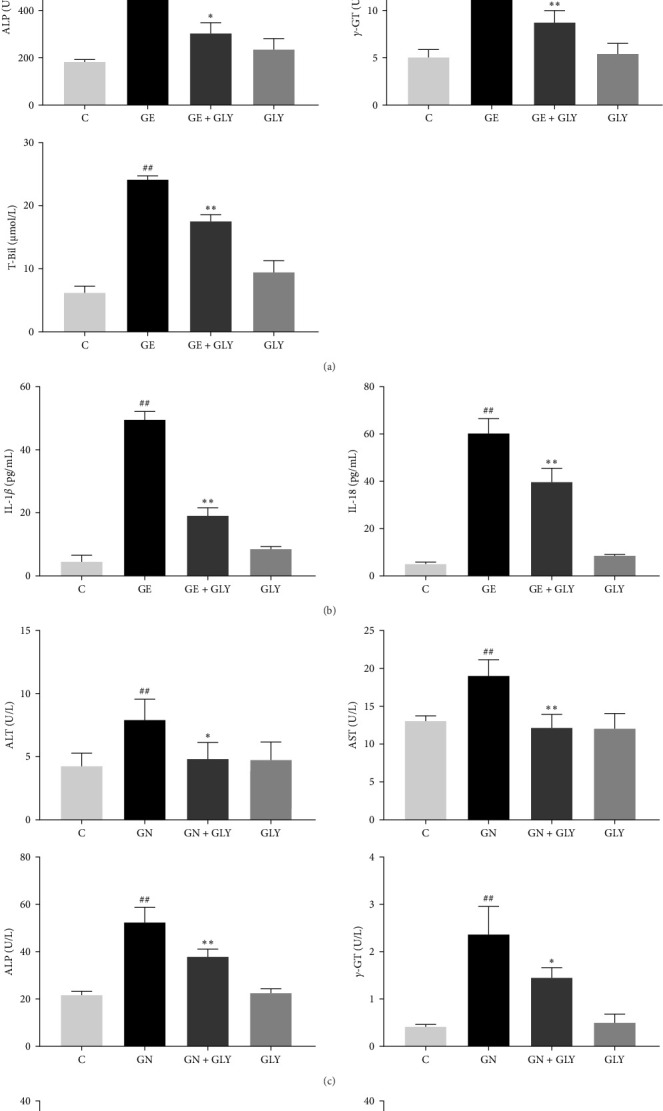
(A) The levels of ALT, AST, ALP, and *γ*-GT in rats treated with geniposide; *n* = 10. (B) The levels of IL-1*β* and L-18 in rats treated with geniposide; *n* = 10. (C) The levels of ALT, AST, ALP, and *γ*-GT in the cell supernatant treated with genipin; *n* = 3. (D) The levels of IL-1*β* and L-18 in the cellsupernatant treated with genipin.*n* = 3 (^#^*p* < 0.05, ^##^*p* < 0.01, vs., control group; *⁣*^*∗*^*p* < 0.05, *⁣*^*∗∗*^*p* < 0.01, vs., geniposide or genipin group). ALP, alkaline phosphatase; ALT, alanine aminotransferase; AST, aspartate aminotransferase; GLY, glibenclamide.

The administration of geniposide led to a marked elevation in the levels of ALT, AST, ALP, *γ*-GT, T-Bil, IL-1*β*, and IL-18 when compared to the control group. However, concurrent administration of GLY with geniposide resulted in a reduction of these markers relative to the geniposide-only group. Interestingly, the consumption of GLY alone caused an increase in T-Bil levels compared to the control group, potentially associated with the drug's hepatic metabolic processes (Figures 2A,B). Moreover, the levels of ALT, AST, ALP, *γ*-GT, IL-1*β*, and IL-18 in the supernatant significantly rose following genipin treatment compared to the control group. Nevertheless, when GLY was coadministered with genipin, these molecules showed a decrease in levels compared to the genipin-only group (Figures 2C,D).

### 3.3. H&E-Stained Sections

**Figure 3:**
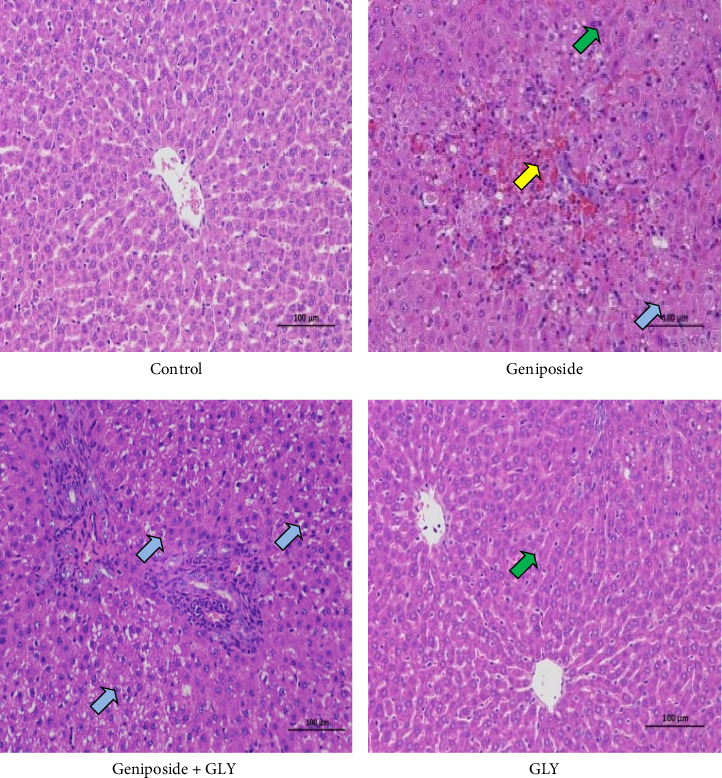
H&E staining results of rats liver (200x). Yellow arrow—infiltration of inflammatory cells with necrosis of hepatocytes. Green arrow—cell steatosis. Blue arrow—cytoplasmic eosinophilic enhancement. AST, aspartate aminotransferase; GLY, glibenclamide.

In the control group, the liver tissue displayed an intact lobular structure with regular hepatocyte arrangement, clear demarcation, and normal cell morphology. In the GLY group, there was a slight increase of bile ducts around the portal area, along with mild hepatocyte steatosis and the presence of small round vacuoles in the cytoplasm. This may be attributed to the inhibition effect of bile salt export pump (BSEP, ABC11) [19]. The geniposide group exhibited disrupted tissue lobules, reduced hepatocyte count, extensive hepatocyte necrosis, hyperplasia of bile ducts around the portal area, pyknotic or fragmented and dissolved nuclei, intensified cytoplasmic eosinophilia, multiple hemorrhages, and inflammatory cell infiltration. Additionally, many hepatocytes showed fatty degeneration and the presence of tiny round vacuoles in the cytoplasm. In the geniposide + GLY group, hepatocyte steatosis was prevalent, particularly around the central vein and portal area, accompanied by the appearance of small round vacuoles in the cytoplasm and mild lymphocyte infiltration. Occasional eosinophilic bodies were also observed. Comparatively, the geniposide group exhibited evident liver damage, which was mitigated to varying degrees after the administration of GLY (Figure 3).

### 3.4. NLRP3 Levels in the Liver and Cells

**Figure 4:**
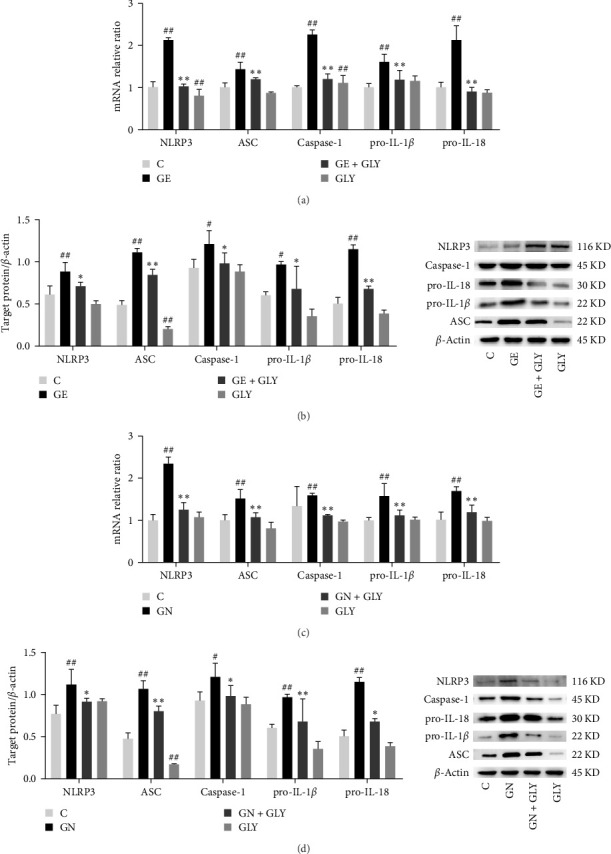
(A) The mRNA levels of NLRP3, ASC, caspase-1, pro-IL-1*β*, pro-IL-18 in the liver of rats treated with geniposide; *n* = 3. (B) The protein levels of NLRP3, ASC, caspase-1, pro-IL-1*β*, pro-IL-18 in the liver of rats treated with geniposide; *n* = 3. (C) The mRNA levels of NLRP3, ASC, caspase-1, pro-IL-1*β*, pro-IL-18 in the cell treated with genipin; *n* = 3. (D) The protein levels of NLRP3, ASC, caspase-1, pro-IL-1*β*, pro-IL-18 in the cell treated with genipin; *n* = 3 (^#^*p* < 0.05, ^##^*p* < 0.01, vs., control group; *⁣*^*∗*^*p* < 0.05, *⁣*^*∗∗*^*p* < 0.01, vs., geniposide or genipin group). ASC, apoptosis-associated speck-like protein; GLY, glibenclamide.

Given the close relationship between NLRP3 inflammasome, IL-1*β*, and IL-18 and their role in amplifying inflammatory reactions and liver disease progression, we assessed the mRNA and protein levels of NLRP3 inflammasome in liver tissues and cells to explore the connection between geniposide-induced hepatotoxicity and NLRP3 inflammasome. As illustrated in Figure 4A,B, RT-PCRs revealed significant increases in the levels of NLRP3, ASC, caspase-1, pro-IL-1*β*, and pro-IL-18 following geniposide treatment. Subsequently, the administration of the NLRP3 inhibitor GLY resulted in a reduction in the mRNA expression of NLRP3, ASC, caspase-1, pro-IL-1*β*, and pro-IL-18 compared to the geniposide group. Moreover, the geniposide group exhibited higher expressions of NLRP3, ASC, caspase-1, pro-IL-1*β*, and pro-IL-18 than the control group. However, following GLY treatment, the protein expression levels of NLRP3, ASC, caspase-1, pro-IL-1*β*, and pro-IL-18 were significantly diminished. In parallel, upon treatment of L02 cells with genipin, the mRNA expressions of NLRP3, ASC, caspase-1, pro-IL-1*β*, and pro-IL-18 increased compared to the control group. These mRNA expressions decreased after GLY administration, in contrast to the genipin group (Figure 4C). Further investigations into protein expression in cells confirmed varying degrees of upregulation of NLRP3-related proteins after genipin treatment, which subsequently decreased following GLY administration (Figure 4D). The results from in vitro and in vivo experiments collaboratively suggest a potential link between geniposide-induced inflammasome activation and NLRP3.

## 4. Discussion

Geniposide, a well-defined major active component isolated from the fruit of the traditional medicinal herb *G. jasminoides* Ellis, exhibits significant anti-inflammatory, antiapoptotic, and antifibrotic properties. It has been applied in various medical fields, notably in the treatment of liver disorders [4]. Nonetheless, it is an indisputable fact that geniposide can cause considerable liver toxicity. The potential for geniposide to induce liver toxicity is attributed to several mechanisms, including the disruption of purine and pyrimidine metabolism [20, 21], changes in bile acid metabolism [11], and disturbances in amino acid metabolism [22]. Additionally, it may contribute to oxidative stress [23], activate the nuclear factor kappa-light-chain-enhancer of activated B cells (NF-*κ*B) pathway [24], and provoke inflammatory responses. Consequently, elucidating the mechanism of its hepatotoxicity and ensuring its safety are crucial for its widespread application. This study investigated changes in the NLRP3 pathway in rat liver toxicity caused by geniposide and in cell toxicity caused by genipin. The findings, for the first time, unequivocally demonstrate a close correlation between geniposide-induced hepatotoxicity and the activation of the NLRP3 pathway. Furthermore, the administration of an NLRP3 inhibitor significantly alleviated the toxic effects.

Following administration, serum and liver tissue samples were collected for liver function-related assessments. The levels of ALT, AST, ALP, *γ*-GT, and T-Bil were significantly elevated in the geniposide group. AST is predominantly localized in hepatocyte cytoplasm, while ALT is mainly distributed in hepatocyte cytoplasm and mitochondria. These enzymes serve as biomarkers of liver function, with increased ALT levels indicating damage to the hepatocyte membrane and elevated AST indicating damage to these organelles. ALP and *γ*-GT are enzymes found in various organisms, especially in organs such as the liver. The main function of *γ*-GT in organisms is to participate in the metabolic process of glutathione, helping the body convert substances such as glutamine into glutathione. ALP can be excreted outside the gallbladder through the liver. T-Bil is a significant product in bile metabolism and is closely associated with various diseases, including hepatitis and jaundice. Furthermore, FG is utilized in the treatment of cholestatic hepatitis, as exemplified by Yinchenhao decoction [25]. Research indicates that geniposide can modulate bile acid levels through farnesoid X receptor (FXR) pathways [26]. Previous research has also highlighted that this modulation exhibits a dual nature; specifically, low doses of geniposide confer a hepatoprotective effect by promoting the excretion of bile acids and maintaining bile acid homeostasis. Conversely, when administered at high doses, geniposide can induce cholestasis and disrupt bile acid biosynthesis, which may lead to hepatotoxicity [27]. In this study, elevated levels of T-Bil further corroborate that high-dose geniposide can result in disturbances in intrahepatic bile acid metabolism. Consequently, the aforementioned indicators are frequently employed as metrics for assessing liver health in clinical evaluations. The increase in these indicators in the study suggests that liver function is affected. Additionally, histological examination of liver tissues through H&E staining revealed evident immuno-inflammation alongside liver damage, suggesting that geniposide-induced hepatotoxicity may be associated with inflammation.

The NLRP3 inflammasome functions as the primary sensor for inflammatory signals and plays a crucial role in initiating inflammatory responses across a range of diseases. An expanding body of evidence highlights the central role of NLRP3 inflammasomes in the development of numerous liver conditions, encompassing alcoholic and nonalcoholic fatty liver diseases, as well as liver injury [12, 28–31]. NLRP3 interacts with pro-caspase-1 and an ASC in the cytoplasm to assemble into an inflammasome [32, 33]. Activation of NLRP3 results in caspase-1 cleavage and the maturation of interleukin IL-18 and IL-1*β*, which play critical roles in driving liver inflammation. Notably, common drugs like carbamazepine and acetaminophen have been demonstrated to induce NLRP3 inflammasome activation, leading to hepatotoxicity [34–38]. Additionally, prior studies have indicated that certain traditional Chinese medicines, such as Triptolide, possess the ability to induce HILI, resulting in NLRP3 inflammasome activation [39, 40]. Given the close relationship between NLRP3 inflammasome, IL-1*β*, and IL-18 and their pivotal roles in the progression of liver diseases through the recruitment of inflammatory cells, we assessed the expression of NLRP3 inflammasome. The findings indicate heightened expression of NLRP3-related genes and proteins, including ASC, caspase-1, pro-IL-1*β*, and pro-IL-18, in the liver. Simultaneously, the levels of IL-1*β* and IL-18 in the serum significantly increased. Subsequently, we substantiated the involvement of NLRP3 inflammasome in geniposide-induced hepatotoxicity by employing NLRP3 inhibitors (GLY, which inhibits K^+^efflux). The heightened levels of two ILs following geniposide exposure were effectively suppressed by NLRP3 inhibitors. Furthermore, the elevated protein and mRNA levels of NLRP3, ASC, caspase-1, pro-IL-1*β*, and pro-IL-18 induced by geniposide were notably diminished in the presence of these inhibitors. Additionally, both histological (H&E staining) and serological examinations indicated a marked improvement in liver injury. This underscores the significance of NLRP3 inflammasome activation as a pivotal mechanism in geniposide-induced hepatotoxicity.

Moreover, to further investigate the toxic effects of FG on hepatocytes, we chose genipin and the normal liver cell line L02 as our research subjects. Geniposide is known to convert into genipin upon hydrolysis by *β*-glucosidase in the intestine, a process confirmed as the primary cause of geniposide toxicity in the body in previous studies. Thus, we utilized genipin as a reference drug for in vivo and in vitro experiments [22, 41]. Following exposure to genipin, there was a significant increase in the levels of ALT, AST,*γ*-GT, and ALP in the cell supernatant, along with heightened levels of IL-1*β* and IL-18. Subsequently, we assessed the expression of NLRP3, revealing a substantial overexpression of NLRP3-related genes and proteins in the cells. However, upon administration of GLY, this overexpression of NLRP3 in the cells was effectively mitigated, and there was a concurrent reduction in the levels of ALT, AST, *γ*-GT, ALP,IL-18, and IL-1*β* in the cell supernatants. This serves as further corroboration of NLRP3′s involvement in the liver damage induced by geniposide. Furthermore, recent studies have confirmed that reducing NLRP3 expression can protect against liver damage caused by exogenous substances. This study, through both in vitro and in vivo experiments, suggests that inhibiting the overexpression of NLRP3 can mitigate the harm inflicted by geniposide on the liver [42–45].

**Figure 5:**
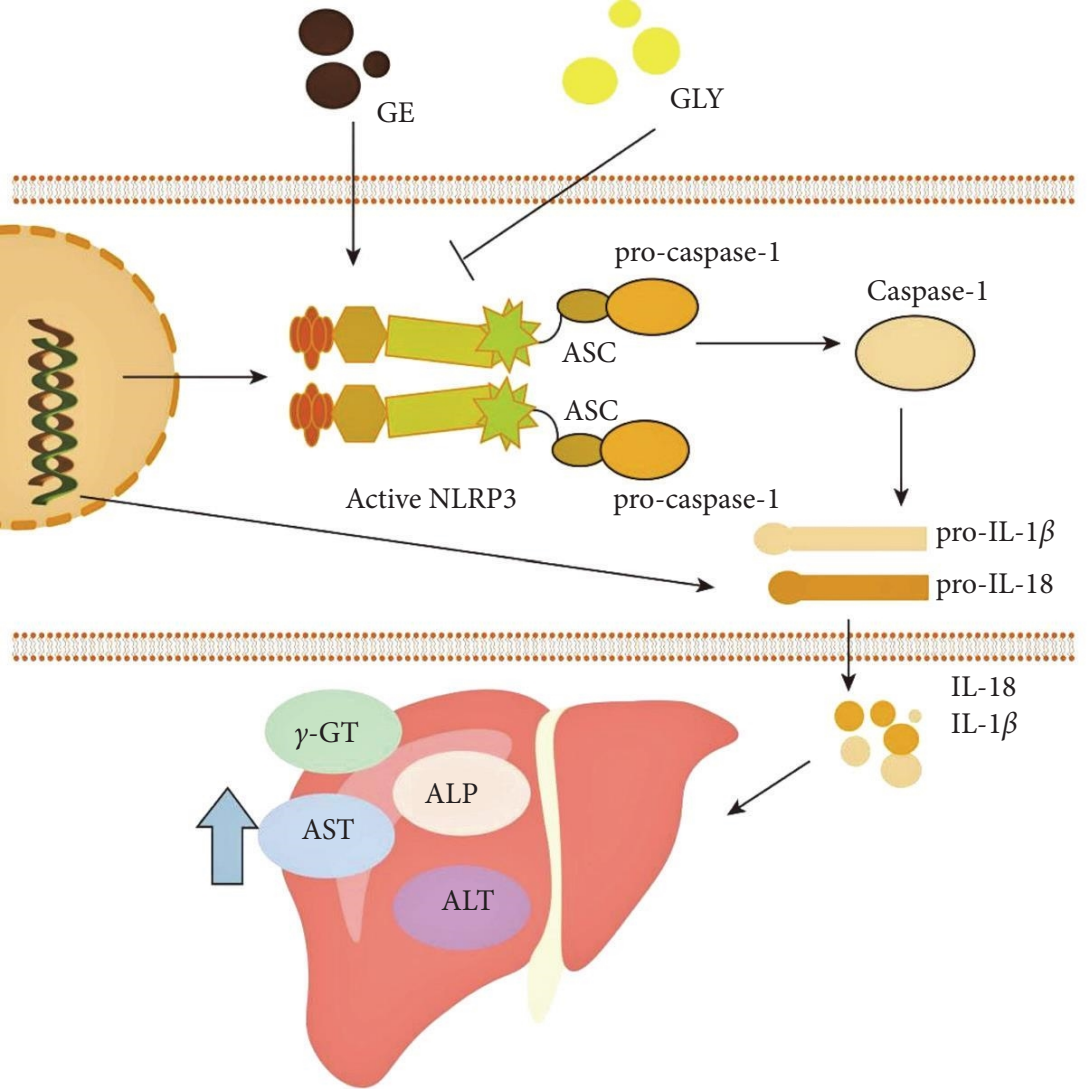
Geniposide induces liver toxicity by activating NLRP3. ALP, alkaline phosphatase; ALT, alanine aminotransferase; ASC, apoptosis-associated speck-like protein; AST, aspartate aminotransferase; GLY, glibenclamide.

In conclusion, as shown in Figure 5, this study elucidates that geniposide can trigger the activation of NLRP3, releasing IL-18 and IL-1*β* to produce inflammatory responses, ultimately leading to liver toxicity. Inhibiting the activation of NLRP3 can reduce the inflammatory response and improve this toxicity.

## Figures and Tables

**Table 1:** Target gene names and quantitative real-time PCR primer sequences for rats.

Target gene	Primer sequence
GAPDH	Forward:5′- AAGAGATGGGAATGTTGGCTG-3′ Reverse: 5′- CTCCCTGCATGACTTTGTTGTC-3′
NLRP3	Forward:5′-TTCCCAGACCCTCATGTTGC-3′
	Reverse: 5′-AGGTCCAGTTCAGTGAGGCT-3′
ASC	Forward:5′-ATTATGGAAGAGTCTGGAGCTGTGG-3′
	Reverse: 5′-TTCTGTGACCCTGGCAATGAGTG-3′
Caspase-1	Forward:5′- TGCCTGGTCTTGTGACTTGGAG -3′
	Reverse: 5′- TGTCCTGGGAAGAGGTAGAAACG -3′
pro-IL-1*β*	Forward:5′-GGGATGATGACGACCTGCTA-3′
	Reverse: 5′-CCACTTGTTGGCTTATGTTCTG-3′
pro-IL-18	Forward:5′-GAAGGATGTCTACCCTCTCCTG-3′
	Reverse: 5′-TGTGTCCTGGCACACGTTTC-3′

Abbreviation: ASC, apoptosis-associated speck-like protein.

**Table 2:** Target gene names and quantitative real-time PCR primer sequences for cells.

Target gene	Primer sequence
GAPDH	Forward:5′-CTGGAGAAACCTGCCAAGTATG-3′ Reverse: 5′-GGTGGAAGAATGGGAGTTGCT-3′
NLRP3	Forward:5′-CAAGAGCGGAGAGAAGGTCG-3′
	Reverse: 5′-CTCTCTCGAAGGTCGTGCAG-3′
ASC	Forward:5′-ACAATGACTGTGCTTAGAGACA-3′
	Reverse: 5′-CACAGCTCCAGACTCTTCTTTA-3′
Caspase-1	Forward:5′-TCCTTGTTTCTCTCCACGGC-3′
	Reverse: 5′-AACACCCACTCGTACACGTC-3′
pro-IL-1*β*	Forward:5′-GAACAACAACAATGCCTCGTGC-3′
	Reverse: 5′-GACAAACCGCTGSTTCCATCTTCT-3′
pro-IL-18	Forward:5′-CAGAAGGACTGACCTCGCC-3′
	Reverse: 5′-CAGAAGTACCTGAGCTCGCC-3′

Abbreviation: ASC, apoptosis-associated speck-like protein.

## Data Availability

Data will be made available on request.

## References

[1] Chow H. C., So T. H., Choi H. C. W., Lam K. O. (2019). Literature Review of Traditional Chinese Medicine Herbs-Induced Liver Injury From an Oncological Perspective With RUCAM. *Integrative Cancer Therapies*.

[2] Efferth T., Kaina B. (2011). Toxicities by Herbal Medicines With Emphasis to Traditional Chinese Medicine. *Current Drug Metabolism*.

[3] Liu H., Chen Y.-F., Li F., Zhang H.-Y. (2013). Fructus Gardenia (Gardenia jasminoides J. Ellis) Phytochemistry, Pharmacology of Cardiovascular, and Safety With the Perspective of New Drugs Development. *Journal of Asian Natural Products Research*.

[4] Chen L., Li M., Yang Z. (2020). *Gardenia jasminoides* Ellis: Ethnopharmacology, Phytochemistry, and Pharmacological and Industrial Applications of an Important Traditional Chinese Medicine. *Journal of Ethnopharmacology*.

[5] National Pharmacopoeia Commission (2020). *Pharmacopoeia of the People’s Republic of China*.

[6] Zhou Y.-X., Zhang R.-Q., Rahman K., Cao Z.-X., Zhang H., Peng C. (2019). Diverse Pharmacological Activities and Potential Medicinal Benefits of Geniposide. *Evidence-Based Complementary and Alternative Medicine*.

[7] Cui Y., Sun R., Wang Q., Wang M. (2017). Hepatotoxicity Induced by Intragastrically Administrated With Gardenia Decoction in Mice. *Natural Product Research*.

[8] Wang Y., Feng F. (2019). Evaluation of the Hepatotoxicity of the Zhi-Zi-Hou-Po Decoction by Combining UPLC-Q-Exactive-MS-Based Metabolomics and HPLC-MS/MS-Based Geniposide Tissue Distribution. *Molecules*.

[9] Tian J., Yi Y., Zhao Y. (2018). Oral Chronic Toxicity Study of Geniposide in Rats. *Journal of Ethnopharmacology*.

[10] Khanal T., Kim H. G., Choi J. H. (2012). Biotransformation of Geniposide by Human Intestinal Microflora on Cytotoxicity Against HepG2 Cells. *Toxicology Letters*.

[11] Tian J., Zhu J., Yi Y. (2017). Dose-Related Liver Injury of Geniposide Associated with the Alteration in Bile Acid Synthesis and Transportation. *Scientific Reports*.

[12] Szabo G., Csak T. (2012). Inflammasomes in Liver Diseases. *Journal of Hepatology*.

[13] Man S. M., Karki R., Sasai M. (2016). IRGB10 Liberates Bacterial Ligands for Sensing by the AIM2 and Caspase-11-NLRP3 Inflammasomes. *Cell*.

[14] Lu A., Magupalli V. G., Ruan J. (2014). Unified Polymerization Mechanism for the Assembly of ASC-Dependent Inflammasomes. *Cell*.

[15] Strowig T., Henao-Mejia J., Elinav E., Flavell R. (2012). Inflammasomes in Health and Disease. *Nature*.

[16] Ke B., Shen W., Fang X., Wu Q. (2018). The NLPR3 Inflammasome and Obesity-Related Kidney Disease. *Journal of Cellular and Molecular Medicine*.

[17] Sano M., Komiyama H., Shinoda R. (2022). NLRP3 Inflammasome Is Involved in Testicular Inflammation Induced by Lipopolysaccharide in Mice. *American Journal of Reproductive Immunology*.

[18] El-Kharashi O. A., El-Din Aly El-Waseef D. A., Nabih E. S., Mohamed D. I. (2018). Targeting NLRP3 Inflammasome via Acetylsalicylic Acid: Role in Suppressing Hepatic Dysfunction and Insulin Resistance Induced by Atorvastatin in Naïve Versus Alcoholic Liver in Rats. *Biomedicine & Pharmacotherapy*.

[19] Liu H., Irobalieva R. N., Kowal J. Structural Basis of Bile Salt Extrusion and Small-Molecule Inhibition in Human BSEP. *Nature*.

[20] Zhao J., Xie C., Mu X. (2018). Metabolic Alterations in Triptolide-Induced Acute Hepatotoxicity. *Biomedical Chromatography*.

[21] Shi W., Jiang Y., Zhao D. S. (2020). Metabolomic-Transcriptomic Landscape of 8-Epidiosbulbin E Acetate -a Major Diterpenoid Lactone From *Dioscorea bulbifera* Tuber Induces Hepatotoxicity. *Food and Chemical Toxicology*.

[22] Li Y., Pan H., Li X. (2019). Role of Intestinal Microbiota-Mediated Genipin Dialdehyde Intermediate Formation in Geniposide-Induced Hepatotoxicity in Rats. *Toxicology and Applied Pharmacology*.

[23] Xiao X., Hu Q., Deng X. (2022). Old Wine in New Bottles: Kaempferol Is a Promising Agent for Treating the Trilogy of Liver Diseases. *Pharmacological Research*.

[24] Mulero M. C., Huxford T., Ghosh G. (2019). NF-κB,I*κ*B, and IKK: Integral Components of Immune System Signaling. *Advances in Experimental Medicine and Biology*.

[25] Luo S., Huang M., Lu X. (2024). Optimized Therapeutic Potential of Yinchenhao Decoction for Cholestatic Hepatitis by Combined Network Meta-Analysis and Network Pharmacology. *Phytomedicine*.

[26] Keitel V., Dröge C., Häussinger D. (2019). Targeting FXR in Cholestasis. *Handbook of Experimental Pharmacology*.

[27] Luo Y., Gao F., Chang R. (2021). Metabolomics Based Comprehensive Investigation of Gardeniae Fructus Induced Hepatotoxicity. *Food and Chemical Toxicology*.

[28] Abdel-Gaber S. A., Geddawy A., Moussa R. A. (2019). The Hepatoprotective Effect of Sitagliptin Against Hepatic Ischemia Reperfusion-Induced Injury in Rats Involves Nrf-2/HO-1 Pathway. *Pharmacological Reports*.

[29] Wree A., McGeough M. D., Peña C. A. (2014). NLRP3 Inflammasome Activation Is Required for Fibrosis Development in NAFLD. *Journal of Molecular Medicine*.

[30] Petrasek J., Bala S., Csak T. (2012). IL-1 Receptor Antagonist Ameliorates Inflammasome-Dependent Alcoholic Steatohepatitis in Mice. *Journal of Clinical Investigation*.

[31] Dixon L. J., Flask C. A., Papouchado B. G., Feldstein A. E., Nagy L. E. (2013). Caspase-1 as a Central Regulator of High Fat Diet-Induced Non-Alcoholic Steatohepatitis. *PLoS ONE*.

[32] Bruchard M., Rebé C., Derangère V. (2015). The Receptor NLRP3 Is a Transcriptional Regulator of TH2 Differentiation. *Nature Immunology*.

[33] Sharma B. R., Kanneganti T.-D. (2021). NLRP3 Inflammasome in Cancer and Metabolic Diseases. *Nature Immunology*.

[34] Kamel E. O., Hassanein E. H. M., Ahmed M. A., Ali F. E. M. (2020). Perindopril Ameliorates Hepatic Ischemia Reperfusion Injury Via Regulation of NF-*κ*B-p65/TLR-4, JAK1/STAT-3, Nrf-2, and PI3K/Akt/mTOR Signaling Pathways. *The Anatomical Record*.

[35] Yang J., Zhu A., Xiao S. (2019). Anthraquinones in the Aqueous Extract of Cassiae Semen Cause Liver Injury in Rats Through Lipid Metabolism Disorder. *Phytomedicine*.

[36] Zhang C., Feng J., Du J. (2018). Macrophage-Derived IL-1*α* Promotes Sterile Inflammation in a Mouse Model of Acetaminophen Hepatotoxicity. *Cellular & Molecular Immunology*.

[37] Zhang X., Luan J., Chen W. (2018). Mesoporous Silica Nanoparticles Induced Hepatotoxicity via NLRP3 Inflammasome Activation and Caspase-1-Dependent Pyroptosis. *Nanoscale*.

[38] Shi L., Zhang S., Huang Z. (2020). Baicalin Promotes Liver Regeneration After Acetaminophen-Induced Liver Injury by Inducing NLRP3 Inflammasome Activation. *Free Radical Biology and Medicine*.

[39] Wang Z., Xu G., Zhan X. (2019). Carbamazepine Promotes Specific Stimuli-Induced NLRP3 Inflammasome Activation and Causes Idiosyncratic Liver Injury in Mice. *Archives of Toxicology*.

[40] Yuan Z., Hasnat M., Liang P. (2019). The Role of Inflammasome Activation in Triptolide-Induced Acute Liver Toxicity. *International Immunopharmacology*.

[41] Wang S., Ge S., Chen Y. (2023). Acute and Subacute Hepatotoxicity of Genipin in Mice and Its Potential Mechanism. *Heliyon*.

[42] Li C., Lan M., Lv J. (2019). Screening of the Hepatotoxic Components in Fructus Gardeniae and Their Effects on Rat Liver BRL-3A Cells. *Molecules*.

[43] Ruan S., Yang Y., Li W. (2022). Antrodia Camphorata Polysaccharide Activates Autophagy and Regulates NLRP3 Degradation to Improve Liver Injury-Related Inflammatory Response. *Aging*.

[44] Xiao L., Qi L., Zhang G. (2022). *Polygonatum sibiricum* Polysaccharides Attenuate Lipopoly-Saccharide-Induced Septic Liver Injury by Suppression of Pyroptosis via NLRP3/GSDMD Signals. *Molecules*.

[45] Liu Y., Liu N., Liu Y. (2022). Ginsenoside Rb1 Reduces D-GalN/LPS-Induced Acute Liver Injury by Regulating TLR4/NF-*κ*B Signaling and NLRP3 Inflammasome. *Journal of Clinical and Translational Hepatology*.

